# Health-Related Quality of Life in Women With Invasive Ductal Carcinoma After Chemotherapy: Insight From the FACT-B+4 Questionnaire

**DOI:** 10.7759/cureus.91785

**Published:** 2025-09-07

**Authors:** Sheela Pavithran, Keechilat Pavithran, Manu Raj, A Anand Kumar, Siby Gopinath

**Affiliations:** 1 Amrita College of Nursing, Amrita Institute of Medical Sciences, Amrita Vishwa Vidyapeetham, Kochi, IND; 2 Medical Oncology, Amrita Institute of Medical Sciences, Amrita Vishwa Vidyapeetham, Kochi, IND; 3 Paediatric Cardiology/Health Sciences Research, Amrita Institute of Medical Sciences, Amrita Vishwa Vidyapeetham, Kochi, IND; 4 Neurology, Amrita Institute of Medical Sciences, Amrita Vishwa Vidyapeetham, Kochi, IND

**Keywords:** breast cancer, chemotherapy, chemotherapy-induced peripheral neuropathy, contributing factors, dose reduction, fact-b+4, health-related quality of life (hrqol), invasive ductal carcinoma, prevalence, women

## Abstract

Introduction

Invasive ductal carcinoma (IDC) is the most common form of breast cancer in females, often treated with chemotherapy, either in the adjuvant or neoadjuvant setting. Malignancy as well as chemotherapy have a profound impact on the quality of life (QOL), with prognosis and survival largely dependent on early identification and treatment. Understanding the determinants of QOL is crucial to addressing its impact on physical, emotional, and social well-being.

Aim

This study aimed to determine the health-related quality of life (HRQOL) of patients with invasive ductal carcinoma after receiving chemotherapy and to explore factors contributing to a reduction in their HRQOL.

Methods

This was a prospective cohort study conducted among 294 women aged between 30 and 70 years who were histopathologically diagnosed with IDC and received chemotherapy from a Comprehensive Cancer Centre, Kerala. QOL was assessed using the Functional Assessment of Cancer Therapy-Breast+4 (FACT-B+4) questionnaire version 4.0, and sociodemographic and clinical data were collected through questionnaires, direct measurements, and from electronic medical records (EMR). The Mann-Whitney U test was used to compare the reduction (change) in QOL, and multiple logistic regression analysis was used to explore the factors contributing to the reduction in QOL after chemotherapy.

Results

The patients had a mean age of 51.56 ± 10.06 years, with the majority being 50 years and above (56.4%). Of 294 women who received chemotherapy, 228 (77.6%) developed chemotherapy-induced peripheral neuropathy (CIPN) of mild to moderate grades. Emotional well-being (EWB) had the lowest mean score before starting chemotherapy (those who developed CIPN: 14.46 ± 5.07 and those who did not develop CIPN: 13.72 ± 4.73). After chemotherapy, the maximum reduction in QOL occurred in physical well-being (PWB), with mean scores of 9.49 ± 5.57 and 3.49 ± 5.62, respectively, for those who developed and did not develop CIPN. Furthermore, in all domains, the reduction in QOL was greater in patients who developed CIPN than in those who did not. However, the difference in QOL change between the two groups was significant only for two domains: PWB (p < 0.001) and EWB (p = 0.013). The breast cancer subscale (BCS) (p < 0.001), arm (ARM) subscale (p = 0.033), and total scores (FACT-B TOI, FACT-G Total, and FACT-B Total) were also significantly lower in women who developed CIPN than in those who did not (p < 0.001). The factors contributing to the reduction in QOL were the presence of CIPN (OR = 3.623, 95% CI: 1.072-12.228, p = 0.038), age <50 years (OR = 4.016, CI: 1.236-12.987, p = 0.007), a higher number of chemotherapy cycles (eight cycles - OR = 38.488, 95% CI: 2.086-710.055, p = 0.053 and 12 cycles - OR = 70.655, 95% CI: 2.067-2415.638, p = 0.018), metastasis in the ipsilateral lymph nodes (OR = 3.623, CI: 1.204-10.902, p = 0.022), use of statins (OR = 7.608, CI: 1.269-45.617, p = 0.026), endocrine drugs (OR = 14.073, CI: 2.180-90.864, p = 0.005), and anaemia (OR = 30.606, CI: 1.867-15.001, p = 0.008).

Conclusion

The study highlights the significant impact of CIPN on the QOL of patients, along with factors such as age, number of chemotherapy cycles, presence of regional metastasis, histopathological subtypes, and anaemia. The study findings suggest the need for risk stratification and tailoring treatment strategies appropriately to minimise the risk of CIPN and optimise the QOL.

## Introduction

Breast, cervical, ovarian, uterine, and vulvar cancers in females are a significant global public health concern [1,2]. Breast cancer is the most common cancer among women in 2022, and cervical cancer is a substantial contributor to gynaecological cancer cases and deaths worldwide. The incidence of cervical cancer is expected to drop by 2040, while that of breast cancer is expected to double [3]. Women often hesitate to disclose warning signs due to stigma, fear about the diagnosis, or lack of awareness, leading to diagnosis and treatment at an advanced stage of the disease, where treatment or cure is limited. Unlike cervical cancer, no vaccine or preventive strategy is available for breast cancer. Hence, screening and controlling risk factors are the major steps for early detection and treatment of breast cancer in its early stages.

Breast cancer data extracted from GLOBACON 2022 across 21 United Nations (UN) regions and 185 countries projected an estimate of 2.3 million new breast cancer cases and 666,000 breast cancer-related deaths in women. The Eastern Asian region reported the highest number of cases, whereas the South-Central Asian region had the highest number of deaths. China had the highest number of breast cancer cases, while India had the highest number of deaths at the country level. If the present rates remain stable, breast cancer cases will increase by 54.7% and deaths by 70.9% by 2050 [4]. Infiltrating ductal carcinoma (IDC) is the most common histopathological breast cancer subtype globally [5,6], accounting for 80% of all breast malignancies [5,7]. It is also the most common histological subtype of breast cancer in India and among men worldwide. Breast cancer, particularly IDC, is a leading cause of cancer-related deaths among Indian women and is more invasive and less aggressive than medullary breast carcinoma subtypes [8].

The risk factors for IDC include genetic factors such as mutations in the breast cancer genes BRCA 1 and BRCA 2; advancing age; menarche before 12 years; menopause after 55 years; first pregnancy after 30 years; those who have not breastfed or ever had children; increase in breast density; personal history of breast cancer; history of benign breast diseases; and history of radiation to the chest area [9,10]. IDC may be asymptomatic in the early stages, a reason for late diagnosis, and a substantial increase in mortality rate at the global level [6]. This warrants regular screening and self or clinical breast examinations. Symptoms, if present, include a lump or mass in the breast felt during self or clinical breast examinations; changes in the size, shape, or contour of the breast; and skin changes such as scaly or flaky skin on the nipple or breast [9,10]. The prognosis depends on the stage at which it is identified and treated. Early identification has been attributed to a high survival rate.

The treatment modalities for IDC include surgery, radiation therapy, and systemic therapies such as chemotherapy, targeted therapy, hormone therapy, and immunotherapy [9-11]. Patients may undergo different combinations of treatment, and the treatment options vary depending on the stage of the disease and its characteristics, such as hormone receptor status. Chemotherapy is widely used to treat breast cancer and is administered before surgery (neoadjuvant chemotherapy) to shrink the tumour or after surgery (adjuvant chemotherapy) to remove any cancer cells that have been left behind and reduce the risk of recurrence. Although chemotherapy is effective in treating breast cancer, it is a double-edged sword with devastating adverse effects and impacts on the quality of life (QOL). The adverse effects of chemotherapy include hair loss [12-14], nausea and vomiting, anorexia [13-17], oral mucositis, constipation or diarrhoea [13,14], changes in taste [18], chemotherapy-induced peripheral neuropathy (CIPN) [13,19], neutropenia, thrombocytopenia, anaemia [14], fatigue, skin and nail changes [14], and sexual dysfunction [13]. Adverse reactions depend on the chemotherapeutic agent, cumulative dosage, and chemotherapy cycles.

Chemotherapy affects the QOL of patients [16,20,21], and various studies have investigated the effect of chemotherapy on QOL and have addressed treatment-related adverse toxicities. A systematic review reported that chemotherapy significantly reduced health-related quality of life (HRQOL) during treatment [20]. QOL is a significant determinant of the ability to adhere to the chemotherapy treatment schedule, treatment outcomes, and ability to manage symptoms. As the global prevalence of breast cancer continues to rise, it is essential to address the complex interplay between breast cancer, its treatment, and QOL and also to develop effective strategies to mitigate the impact of this disease. The current study was conducted with the objective of determining the HRQOL of patients with IDC treated with chemotherapy and to explore the factors contributing to the reduction in HRQOL. Therefore, the FACT-B+4 questionnaire, a widely used tool to assess the QOL of patients with breast cancer, was used in the study.

## Materials and methods

Study setting and population

This was a secondary analysis of a prospective cohort study conducted among women with breast cancer receiving chemotherapy to estimate the prevalence of CIPN and determine their QOL. The study was conducted at the Comprehensive Cancer Centre, Kerala, between October 2021 and July 2024. The participants were selected based on histopathological reports. Women aged 30-70 years, with anatomical stages I-III, were included in the study. Patients who had received prior radiation, who were receiving palliative care, and who had peripheral neuropathy were excluded. This analysis focused on women with IDC. Data concerning 294 women diagnosed with IDC were extracted from the main study and subjected to analysis.

Data collection instruments and measurements

Data were collected using sociodemographic and clinical data profiles developed by the investigators and the FACT-B+4 questionnaire developed by FACIT.org. FACT-B+4 is widely used for assessing the QOL of patients with breast cancer. The instrument combines the general cancer-related QOL assessment (FACT-G) with breast cancer subscales (BCS) and four additional items to assess arm (ARM) mobility. FACT-B+4, a self-administered questionnaire, includes 29 items of FACT-G that measure physical, social, emotional, and functional well-being (FWB), and a 9-item BCS. The +4 items specifically address the impact of arm-related issues, such as swelling/lymphoedema following breast cancer surgery. The items are scored on a 5-point Likert scale (0-4), with higher scores indicating higher QOL. The instrument provides scores for the individual subscales of physical well-being (PWB), social/family well-being (SWB), emotional well-being (EWB), FWB, BCS, the ARM subscale, and total scores. The tool is available in 29 languages, including Malayalam, and has been validated in various populations worldwide, with reports of its reliability and validity [22]. The Malayalam version of the tool, which all the patients preferred, was used in the study. HRQOL was assessed before the first cycle and on completion of the last cycle of chemotherapy. The data concerning participants who completed all scheduled cycles of chemotherapy only were included in the study. Formal permission was obtained from the tool developers to use the tool for the study.

Statistical analysis

The data analysis was performed using SPSS version 30.0 (IBM Corp., Armonk, NY, USA). Categorical variables were presented as frequencies and percentages, and continuous variables were summarised using means and standard deviations. The Kolmogorov-Smirnov test assessed the normality of the data, which reported non-normal distribution. Hence, the Mann-Whitney U test was used to compare reduction (change) in HRQOL. Factors contributing to a reduction in HRQOL were computed using multivariate logistic regression analysis. The level of significance was set at p<0.05.

## Results

Sample characteristics

The prevalence of CIPN in this study was 77.6% (n = 228). Based on this finding, the data analysed is presented in two categories: with CIPN (those who developed CIPN) and without CIPN (those who did not develop CIPN).

The patients had a mean age of 51.56 ± 10.06 years, with the majority (165, 56.4%) aged 50 years and above; over 65% had an above-normal BMI (>25 kg/m²), and the majority were premenopausal (167, 56.8%). Hypertension was common in participants who did not develop CIPN, whereas diabetes was the most common comorbidity among participants who developed CIPN. More than 70% of patients were treated in the adjuvant setting, 58.8% received paclitaxel, and 41.8% received docetaxel. Over half (55.8%) received the dose-dense AC-T regimen (adriamycin + cyclophosphamide - taxol), and over 60% of the patients who developed CIPN received the AC-T regimen. Of the participants who developed CIPN, 90.4% had locally advanced breast cancer. Anaemia was present in 35.1% of women before commencing chemotherapy. Treatment modifications such as dose delay (62, 21.1%) and dose reduction (67, 22.7%) were also reported (Table 1).

**Table 1:** Sociodemographic and clinical variables *Has multiple responses CIPN – chemotherapy-induced peripheral neuropathy; f – frequency; SD – standard deviation; n – number; BMI – body mass index; AC-T – adriamycin + cyclophosphamide - taxol

Variables	Total f (%) 294 (100.0)	Groups	
		With CIPN f (%) 228 (77.6)	Without CIPN f (%) 66 (22.4%)
Age (Mean+SD)	51.5+10.06	51.73+9.94	50.9+10.55
<50	129 (43.9)	98 (43.0)	31 (47.0)
>50	165 (56.1)	130 (57.0)	35 (53.0)
Marital status			
Married	264 (89.8)	201 (88.2)	63 (95.5)
Single/widowed/separated/divorced	30 (10.2)	27 (11.8)	3 (4.5)
BMI (kg/m^2^)	n = 293		
<25	100 (34.1)	76 (33.3)	24 (36.9)
>25	193 (65.9)	152 (66.7)	41 (63.1)
Menstrual status			
Pre-menopausal	167 (56.8)	127 (55.7)	40 (60.6)
Post-menopausal	127 (43.2)	101 (44.3)	26 (39.4)
Breastfeeding (years)			
Not breastfed	22 (7.5)	15 (6.6)	7 (10.6)
Up to 2 years	101 (34.4)	76 (33.3)	25 (37.9)
More than 2 years	171 (58.1)	137 (60.1)	34 (51.5)
Comorbidity*	172 (58.5)	133 (58.3)	39 (59.1)
Hypertension	80 (27.2)	61 (26.8)	19 (28.8)
Diabetes	75 (25.5)	62 (27.2)	13 (19.7)
Drugs for comorbidity*	144 (49.0)	113 (49.6)	31 (47.0)
Cardiovascular	76 (25.9)	59 (25.9)	17 (25.8)
Endocrine	102 (34.7)	82 (36.0)	20 (30.3)
Statins	45 (15.3)	33 (14.5)	12 (18.2)
Anatomical stage			
Early stage (I & II)	33 (11.2)	22 (9.6)	11 (16.7)
Locally advanced stage (III)	261 (88.8)	206 (90.4)	55 (83.3)
Line of chemotherapy			
Neoadjuvant	116 (39.5)	99 (43.4)	17 (25.8)
Adjuvant	178 (70.5)	129 (56.6)	49 (74.2)
Chemotherapy regimen			
AC – Taxol (Dose-dense)	164 (55.8)	138 (60.5)	26 (39.4)
Others	130 (44.2)	90 (39.5)	40 (60.6)
Taxane agent received*			
Paclitaxel	173 (58.8)	145 (63.6)	28 (42.4)
Docetaxel	123 (41.8)	85 (37.3)	38 (57.6)
Both paclitaxel and docetaxel	2 (0.8%)	2 (0.9)	0 (0.0)
Anemia	n = 287		
Yes	103 (35.0)	80 (35.1)	23 (34.8)
No	184 (62.6)	145 (63.6)	39 (59.1)
Missing	7 (2.4)	3 (1.3)	4 (6.1)
Treatment modification*			
Dose delay	62 (21.1)	52 (22.8)	10 (15.2)
Dose reduction	67 (22.7)	58 (25.4)	9 (13.6)

Of the 294 participants, only 14 (4.8%) were diagnosed through mammography as part of routine screening or as a concern due to family history. For those who presented with symptoms, a lump in the breast was the most common symptom (262, 89.1%). Breast pain (76, 25.9%), painful swelling or oedema in the breast (65, 22.1%), metastasis in the ipsilateral lymph nodes (192, 65.3%), and nipple retraction (42, 14.3%) were also reported (Appendix 1).

Before commencement of chemotherapy, both the groups had the lowest mean (SD) score in EWB (with CIPN: 14.46 (5.07) and without CIPN: 13.72 (4.73)), while SWB had the highest mean (SD) score (with CIPN: 23.89 (3.48) and without CIPN: 24.43 (2.84)). After the chemotherapy, the QOL had declined in all the subscales except for the ARM subscale, which had a slight increase in score (0.04 and 0.79 for with and without CIPN, respectively). Following the completion of chemotherapy cycles, PWB showed the highest reduction in QOL in both groups. In all the domains, except EWB, patients who developed CIPN had a low QOL compared to those who did not develop CIPN. A similar reduction in QOL was reflected in all the total scores: FACT-B TOI, FACT-G Total, and FACT-B Total for the two groups, where those who developed CIPN had a low mean (SD) QOL score. Overall, patients who developed CIPN had a greater decline in QOL in all domains except for EWB, where patients without CIPN had a slight increase in QOL (Table 2).

**Table 2:** Mean, standard deviation and mean difference in quality of life in FACT-B+4 CIPN – chemotherapy-induced peripheral neuropathy; SD – standard deviation FACT-B TOI – Functional Assessment of Cancer Therapy-Breast Trial Outcome Index; FACT-G – Functional Assessment of Cancer Therapy-General; FACT-B Total - Functional Assessment of Cancer Therapy-Breast Total

FACT B+4 Scales	With CIPN – Mean (SD)				Without CIPN - Mean (SD)		
	Before chemotherapy	After chemotherapy	Mean Difference	Before chemotherapy		After chemotherapy	Mean Difference
Physical Well-Being (PWB)	23.54 (3.4)	14.05 (5.00)	9.49 (5.57)	23.27 (4.0)		19.97 (5.25)	3.49 (5.62)
Social/family Well-Being (SWB)	23.89 (3.48)	21.97 (3.52)	1.92 (3.31)	24.43 (2.84)		22.94 (3.20)	1.49+/-3.32
Emotional Well-Being (EWB)	14.46 (5.07)	13.29 (4.88)	1.16 (5.19)	13.72 (4.73)		14.27 (4.05)	-0.56 (4.40)
Functional Well-Being (FWB)	17.65 (4.13)	15.29 (4.02)	2.36 (4.76)	18.08 (3.58)		16.68 (3.44)	1.39 (3.55)
Breast Cancer Subscale (BCS)	29.03 (4.71)	18.08 (4.87)	10.95 (5.67)	28.79 (4.69)		21.24 (4.42)	7.54 (5.98)
Arm Subscale (ARM)	18.49 (2.23)	18.52 (2.46)	-0.04 (2.89)	18.09 (2.55)		18.88 (2.43)	-0.79 (2.94)
FACT- B TOI	70.22 (9.87)	47.42 (11.96)	22.80 (12.40)	70.14 (10.10)		57.71 (10.46)	12.42 (12.02)
FACT-G Total	79.53 (11.80)	64.6 (13.96)	14.93 (13.15)	79.49 (11.28)		73.69 (11.08)	5.81 (11.15)
FACT-B Total	108.28 (14.44)	94.93 (13.93)	25.88 (16.81)	108.28 (14.44)		94.93 (13.93)	13.35 (15.15)

Patients who developed CIPN had a significant reduction in QOL in physical (p < 0.001) and emotional well-being (p = 0.013). The BCS and total scores (FACT-B TOI, FACT-G Total, and FACT-B Total) also significantly reduced in patients who developed CIPN (p < 0.001). However, EWB slightly improved in those who did not develop CIPN (p = 0.033), although the two groups differed significantly in EWB (Table 3).

**Table 3:** Comparison of the Change in Health-related Quality of Life Before and After Chemotherapy Between Groups using Mann-Whitney U test * p<0.05; **p<0.0001; IQR – Inter quartile range CIPN – chemotherapy-induced peripheral neuropathy; SD – standard deviation FACT-B TOI – Functional Assessment of Cancer Therapy-Breast Trial Outcome Index; FACT-G – Functional Assessment of Cancer Therapy-General; FACT-B Total - Functional Assessment of Cancer Therapy-Breast Total

FACT B+4 Subscales	Total (n = 294)	Groups		p- Value
	Median (IQR)	With CIPN (n = 228)	Without CIPN (n = 66)	
		Median (IQR)	Median (IQR)	
Physical Well-Being (PWB)	-8.0 (-12.0, -4.0)	-10.0 (-13.0, -6.0)	-3.0 (-8.0, 1.0)	<0.001**
Social/Family Well-Being (SWB)	2.0 (0.0, 4.0)	2.0 (0.0, 4.0)	1.0 (-0.25, 3.0)	0.112
Emotional Well-Being (EWB)	-1.0 (-4.0, 3.0)	-2.0 (-4.0, 3.0)	0.0 (-3.0, 4.0)	0.013*
Functional Well-Being (FWB)	-2.0 (-5.0, 1.0)	-2.0 (-6.0, 1.0)	-2.0 (-4.0, 1.0)	0.074
Breast Cancer Subscales (BCS)	-11.0 (-14.0, 6.0)	-11.0 (-15.0, -7.0)	-8.0 (-11.25, -3.75)	<0.001**
Arm Subscales (ARM)	0.0 (-30.0, -12.0)	0.0 (-1.0, 1.0)	0.0 (0.0, 2.25)	0.033*
FACT-B TOI	-21.0 (-30.0, -12.0)	-24.0 (-31.0, -14.0)	-12.0 (-20.25, -5.5)	<0.001**
FACT-G Total	-13.0 (-22.25, -3.0)	-15.5 (-25.0, -6.0)	-4.0 (-11.0, 1.0)	<0.001**
FACT-B Total	-23.17 (-36.25, -11.75)	-27 (-39.0, -13.0)	-12.0 (-20.8, -3.0)	<0.001**

The HRQOL of life of the patients with CIPN and without CIPN did not differ significantly before the commencement of chemotherapy in any of the subscales/domains. However, the HRQOL differed significantly between the two groups after chemotherapy in PWB, SWB, FWB, BCS, FACT-B TOI, FACT-G Total, and FACT-B Total (p < 0.05) (Appendix 2).

Significant variables were subjected to multivariate logistic regression analysis to explore the risk factors for the reduction in the HRQOL.

Multiple logistic regression analysis with the FACT-B total score and the selected variables (Table 4) indicated that patients who developed CIPN had a higher risk of reduction in HRQOL than those who did not (OR = 3.623, 95% CI: 1.072-12.228, p = 0.038). Other risk factors for a reduction in HRQOL included age <50 years (OR = 4.016, CI: 1.236 - 12.987, p = 0.007); number of chemotherapy cycles - eight cycles (OR = 38.488, 95% CI: 2.086 - 710.055, p = 0.014) and twelve cycles (OR = 70.655, 95% CI: 2.067 - 2415.638, p = 0.018); metastasis to the ipsilateral lymph nodes (OR = 3.623, CI: 1.204 - 10.902, p = 0.022); use of the drug statins (OR = 7.608, CI: 1.269 - 45.617, p = 0.026), endocrine drugs, and anaemia (OR = 30.606, CI: 1.867 - 15.001, p = 0.008). 

**Table 4:** Contributing factors of reduction in quality of life following chemotherapy CIPN – chemotherapy-induced peripheral neuropathy; OR – odds ratio; CI – confidence interval

Variables	OR	95% CI for OR		p-value
		Lower	Upper	
With CIPN vs without CIPN	3.623	1.072	12.228	0.038
Age group > 50 years	4.016	1.236	12.987	0.007
Number of chemotherapy cycles				
Four (4)	1			
Six (6)	3.243	0.984	10.683	0.053
Eight (8)	38.488	2.086	710.055	0.014
Twelve (12)	70.655	2.067	2415.638	0.018
Estrogen receptor	0.124	0.025	0.625	0.038
Breast pain	0.199	0.059	0.669	0.009
Metastasis in the ipsilateral lymph nodes	3.623	1.204	10.902	0.022
Cardiovascular drugs	7.608	1.269	45.617	0.026
Endocrine drugs	14.073	2.180	90.864	0.005
Anaemia	3.606	1.867	15.001	0.008

## Discussion

Invasive ductal carcinoma, the most prevalent breast cancer subtype, is a prominent global health issue among females that transcends geographical, cultural, and socioeconomic boundaries. Treatment-related factors, such as adverse effects, treatment-related burdens, and healthcare costs, can exacerbate the condition. Addressing the QOL of patients is crucial for successful treatment and survival. The current study was conducted to determine the health-related quality of life of women with invasive ductal carcinoma undergoing chemotherapy and to explore the factors contributing to a reduction in their HRQOL after chemotherapy.

In this study, 77.6% of patients developed CIPN. The analysis of FACT-B+4 projected that PWB had the maximum reduction after chemotherapy, which is consistent with the results of studies conducted in India [16,23] and Indonesia [24], where the PWB domain had the lowest score. However, other studies [16,24] reported the highest score for EWB, which is contrary to the findings of the current study. In the present study, women had the lowest score in EWB even before the commencement of chemotherapy, which did not reduce after chemotherapy. SWB had the highest scores before and after the chemotherapy. This highlights the support received from family/friends during the disease and treatment. More than 90% of the participants were married and had children, which may have influenced this factor. This finding is supported by Anggraeni et al. [24], Faroughi et al. [25], and Kanyamkandi and Sunderam [26]. FWB had a lower score than PWB and SWB, similar to the findings of Aggraeni et al. [24]. The BCS was significantly reduced after chemotherapy, while the arm subscales (ARM) remained largely unchanged. This could be due to the time elapsed between mastectomy/breast surgery and chemotherapy, resulting in less discomfort from the surgical pain.

Furthermore, compared to those who did not develop CIPN, patients who developed CIPN had a significant reduction in PWB (p < 0.001), SWB (p = 0.018), FWB (p = 0.018), BCS (p < 0.001), and in all the total scores of FACT-B+4 (p < 0.001). The EWB, which had the lowest score before the commencement of chemotherapy, did not decline after chemotherapy but differed significantly between the groups, probably due to the increase in EWB after chemotherapy in the group that did not develop CIPN. The reduction in QOL in this study was associated with CIPN, similar to other studies [7,27]; age <50 years, supported by Guan et al. [7] and Khazi et al. [28], while contradicting the report of an association with advancing age [23]. The number of chemotherapy cycles had a significant association with a reduction in HRQOL. Compared to the patients who received four and six cycles, those who received eight and 12 cycles had a significant reduction in HRQOL, indicating an inverse relationship between the number of cycles and HRQOL. Even though the AC-T treatment regimen was significant in the univariate analysis, it was not significant in the multivariate analysis. 

Metastasis in the ipsilateral lymph nodes, use of statins and endocrine drugs, and the presence of anaemia also significantly contributed to the reduction in HRQOL in this study. Obesity or a higher BMI, pre-existing neuropathy [29], and comorbidities [28] also increase the risk for a decrease in QOL in breast cancer. A significant proportion in this study were above the normal range of BMI. However, in multivariate analysis, the difference was not significant. Patients with pre-existing neuropathy were excluded from the study. Diabetic patients in the study also were free from the symptoms of peripheral neuropathy at baseline. The demographic and clinical characteristics in the study population, including advancing age, above-normal BMI, and family history of breast cancer and any other cancer, were consistent with the risk factors for IDC reported in literature.

Apart from the outcome of the primary objective, the current study highlights the alarmingly low rate of breast cancer detection through screening, where only 4.8% of the participants identified the disease through screening. Despite a family history of cancer (52%) and breast cancer (22.8%), a vast majority (88.8%) of the participants identified the disease at a locally advanced stage. More than one-fourth of the patients had a duration since diagnosis of over six months, the longest being five years. More than 51% of the participants had graduate degrees or above, and none were illiterate. These data underscore the need for health literacy, focusing on the need for awareness of the warning signs of breast cancer, risk factors, and the need for screening and early identification of breast cancer. Efforts should focus on promoting regular screening, particularly among high-risk populations, and addressing barriers such as lack of awareness, fear, stigma, and limited access to healthcare services. By prioritising cancer screening and early detection, it is possible to improve treatment outcomes and reduce the burden of cancer on individuals and communities. Additionally, socioeconomic and geographic disparities significantly influence breast cancer outcomes and QOL. Many women encounter barriers such as financial hardship and limited autonomy in healthcare decisions, leading to delayed care, poorer outcomes, and higher mortality. Empowering women and fostering health literacy are vital to overcoming these gender inequalities.

Strengths and limitations of the study

To the best of our knowledge, no studies have specifically examined the QOL of patients with invasive ductal carcinoma undergoing chemotherapy. This study therefore underscores an important gap in the literature and emphasizes the need for further exploration of this population.

As it was not a longitudinal study, the changes in QOL over a period of time could not be assessed. Also, the different regimens and number of cycles included could limit its generalizability.

## Conclusions

The findings of the study highlight the significant challenges faced by patients with breast cancer before, during, and after treatment, especially with treatment-related adverse events such as chemotherapy-induced peripheral neuropathy, which has a serious impact on health-related quality of life. Patients take it for granted that pain and aches are part of the treatment and tend to underreport unless probed. This lack of awareness needs to be addressed, and patient-reported outcome measures must be given importance to accurately document the problems and adopt appropriate prevention and management strategies to improve the quality of life. Ongoing research on the QOL of breast cancer patients can inform healthcare providers, policymakers, and stakeholders, enhancing care and outcomes and ultimately promoting more equitable, effective, and patient-centered care.

## Appendices

Appendix 1 

**Table 5:** Description of presenting symptoms *Has multiple responses CIPN – chemotherapy-induced peripheral neuropathy; LN – lymph nodes

Presenting Symptoms*	Total (n = 294)	With CIPN (n = 228)	Without CIPN (n = 66)
Mammogram Screening	14 (4.8)	10 (4.4)	4 (6.1)
Presenting symptom	283 (96.3)	220 (96.5)	63 (95.5)
Lump in the breast	262 (89.1)	204 (89.5)	58 (87.9)
Increase in the size of the breast	53 (18.0)	42 (18.4)	11 (16.7)
Breast pain	76 (25.9)	62 (27.2)	14 (21.2)
Paeau d’Orange appearance	10 (3.4)	7 (3.1)	3 (4.5)
Painful swelling or edema	65 (22.1)	55 (24.1)	10 (15.2)
Painless swelling	8 (2.7)	6 (2.6)	2 (3.0)
Itching	7 (2.4)	6 (2.6)	1 (1.5)
Ulceration	5 (1.7)	4 (1.80	1 (1.5)
Skin thickening	16 (5.4)	12 (5.3)	4 (6.1)
Redness in the breast	10 (3.4)	10 (4.4)	0 (0.0)
Dimpling in the breast	11 (3.7)	10 (4.4)	1 (1.5)
Change in the shape	4 (1.4)	3 (1.3)	1 (1.5)
Warts	4 (1.4)	4 (1.8)	0 (0.0)
Nipple retraction	42 (14.3)	37 (16.3)	5 (7.6)
Nipple discharge	12 (4.10	12 (5.3)	0 (0.0)
Axillary node enlargement	24 (8.2)	14 (6.1)	10 (15.2)
Weight loss	10 (3.4)	8 (3.5)	2 (3.0)
Hand symptoms	15 (5.1)	14 (6.1)	1 (1.5)
Fatigue	7 (2.4)	7 93.1)	0 (0.0)
Non-specific symptoms	37 (12.6)	30 (13.2)	7 (10.6)
Metastasis in ipsilateral LN*	192 (65.3)	160 (70.2)	32 (48.5)
Cervical LN	7 (2.4)	6 (2.6)	1 (1.5)
Internal mammary LN	27 (9.2)	24 (10.5)	3 (4.5)
Deep pectoralis	54 (18.4)	45 (19.7)	9 (13.6)
Supraclavicular LN	12 (4.1)	10 (4.4)	2 (3.0)

Appendix 2 

**Table 6:** Comparison of Quality of Life Before and After Chemotherapy Within and Between Groups * p<0.05; **p<0.0001; IQR – Inter quartile range CIPN – chemotherapy-induced peripheral neuropathy; SD – standard deviation FACT-B TOI – Functional Assessment of Cancer Therapy-Breast Trial Outcome Index; FACT-G Total – Functional Assessment of Cancer Therapy-General Total; FACT-B Total - Functional Assessment of Cancer Therapy-Breast Total

FACT B+4 subscales	Measurement	Groups		p- Value (Between groups)
		With CIPN n = 228)	Without CIPN (n=66)	
		Median (IQR)	Median (IQR)	
Physical Well-Being (PWB)	Before	24 (21, 26)	23.5 (21, 27)	0.84
	After	14 (10, 17)	20.5 (15, 24)	<0.001**
	p-value (within group)	<0.001	<0.001	
Social Well-Being (SWB)	Before	25 (22, 26)	25 (22, 27)	0.29
	After	22 (20, 25)	24 (21.75, 25)	0.018*
	p-value (within group)	<0.001	<0.001	
Emotional Well-Being (EWB)	Before	15 (11, 18)	13.5 (11, 17)	0.17
	After	14 (10, 17)	14 (11.75, 17)	0.19
	p-value (within group)	0.001	0.362	
Functional Well-Being (FWB)	Before	18 (15, 21)	18 (16, 20)	0.85
	After	16 (13, 18)	17 (14.75, 18.25)	0.018*
	p-value (within group)	<0.001	0.002	
Breast Cancer Subscales (BCS)	Before	29 (26, 33)	28.5 (27.75, 32.1)	0.53
	After	18 (15, 21)	21 (18.75, 24.25)	<0.001*
	p-value (within group)	<0.001	<0.001	
Arm Subscale (ARM)	Before	20 (17, 20)	19 (17, 20)	0.21
	After	20 (18, 20)	20 (18, 20)	0.194
	p-value (within group)	0.762	0.015	
FACT-B TOI	Before	70 (63, 78)	70.5 (64, 76.4)	0.809
	After	48 (38, 55.75)	57.5 (49, 66)	<0.001**
	p-value (within group)	<0.001	<0.001	
FACT-G Total	Before	80 (72, 87)	78.5 (71, 88.25)	0.736
	After	66 (54.75, 74)	74 (66, 81)	<0.001**
	p-value (within group)	<0.001	<0.001	
FACT-B Total	Before	109 (99, 119.75)	107 (97.75, 118.25)	0.696
	After	84.5 (70, 95.75)	95 (84.5, 105.03)	<0.001**
	p-value (within group)	<0.001	<0.001	
